# Combined chemoradiotherapy showed improved outcome with early-stage HPV-positive oropharyngeal cancers

**DOI:** 10.1186/s12885-022-09515-2

**Published:** 2022-05-07

**Authors:** X. J. David Lu, Emmanuel Jackson, Jason Chew, Sally Nguyen, Jonn Wu, Catherine F. Poh, Eitan Prisman

**Affiliations:** 1 Department of Oral Biological and Medical Sciences, Faculty of Dentistry, University of British Columbia, Vancouver, British Columbia Canada; 2 Department of Integrative Oncology, British Columbia Cancer Research Centre, Vancouver, British Columbia Canada; 3 Division of Neuroradiology, Department of Radiology, Vancouver General Hospital, Vancouver, British Columbia Canada; 4 Division of Otolaryngology - Head and Neck Surgery, Department of Surgery, Vancouver General Hospital, Vancouver, British Columbia Canada; 5 Department of Radiation Oncology, BC Cancer Agency, Vancouver Center, Vancouver, British Columbia Canada; 6 Department of Pathology and Laboratory Medicine, University of British Columbia, Vancouver, British Columbia Canada

**Keywords:** Human papillomavirus, Oropharyngeal squamous cell carcinoma, Staging, Survival analysis, Treatment

## Abstract

**Background:**

The revised 8th Edition American Joint Committee on Cancer (AJCC) Head and Neck Staging Manual distinguishes HPV-mediated from non-HPV-mediated oropharyngeal cancer (OpSCC). The objective was to analyze OpSCC treatment modalities and outcomes.

**Methods:**

A retrospective study of OpSCC patients treated with radiotherapy or chemoradiotherapy between January 1st, 2000, and December 31st, 2008, as identified from the BC Cancer Registry. All patients received treatment at cancer clinics and had at least 5 years follow-up post-treatment. A total of 1259 OpSCC patients were identified. After initial chart reviews, 288 patients were excluded from further analysis and the majority (*n* = 198) was due to not receiving curative treatment. Based on the availability of formalin-fixed, paraffin-embedded (FFPE) tissue, patients were divided into two cohorts: Study Cohort (FFPE available, *n* = 244) and General Cohort (FFPE unavailable, *n* = 727). The Study Cohort was restaged according to AJCC 8th Edition based on p16 immunohistochemistry status. Kaplan-Meier analysis was used to estimate the 5-year overall survival (OS), disease-specific survival (DSS), and locoregional recurrence-free survival (LFS).

**Results:**

Among 971 patients, OpSCC age-adjusted incidence rate was observed to have increased from 2.1 to 3.5 per 100,000 between 2000 and 2008. The General Cohort was relatively older than the Study Cohort (60.1 ± 10.5 vs. 57.3 ± 9.4), but both cohorts were predominantly males (78.3% vs. 76.2%). Amongst the Study Cohort, 77.5% were p16-positive, of whom 98.4% were down staged in the 8th Edition. These early-stage patients showed OS improvement for those treated with chemoradiation, compared to radiation alone (85.8% vs. 73.1%, *p* = 0.05).

**Conclusions:**

OpSCC incidence is increasing in BC. The addition of chemotherapy to radiotherapy may portend a benefit in OS even for early-stage p16-positive OpSCC. Additional research is necessary to assess the safety of treatment de-escalation even among early-stage disease.

**Supplementary Information:**

The online version contains supplementary material available at 10.1186/s12885-022-09515-2.

## Background

Efforts to reduce tobacco use have resulted in a decline of the incidence of head and neck cancer. However, oropharyngeal squamous cell carcinoma (OpSCC) continues to increase [1]. Persistent high-risk human papillomavirus (HR-HPV) infections can lead to cancer development and HPV subtypes 16 and 18 has been recognized to cause OpSCC [2]. In conjunction with an increase in the prevalence of oral HPV in the population [3, 4], an accompanied increase in the incidence of HPV-mediated OpSCC is being observed [5].

HPV-positive OpSCC patients often present with small primary tumors, but high nodal burden, compared to their HPV-negative counterparts [6, 7]. The 7th Edition of the American Joint Committee on Cancer (AJCC) Head and Neck Staging Manual, which stresses the significance of nodal involvement [8], will stage most HPV-positive OpSCC to locally advanced disease. Treatment for OpSCC disease is either primary radiation therapy (RT), with or without chemotherapy, or primary surgery, with or without adjuvant RT and/or chemotherapy. Independent of treatment modality, HPV-positive OpSCC patients have significantly improved outcomes compared to HPV-negative OpSCC [9], which has stimulated trials to study the effect of treatment de-escalation, in the hopes of limiting treatment-induced side effects whilst maintaining locoregional control [10, 11]. In recognition of HPV-positive OpSCC being a distinct clinical entity, the 8th Edition of the AJCC Head and Neck Staging Manual has included HPV status, determined by p16 overexpression, as part of its OpSCC staging [12].

The main objectives of this study were to review the trends of OpSCC patients in British Columbia (BC) and to associate survival outcomes with treatment modality of patients with OpSCC.

## Methods

### Study population – general and study cohorts

Patients with OpSCC referred to the BC Cancer Agency (BCCA) for treatment between January 1st, 2000, and December 31st, 2008, were retrospectively identified from the BC Cancer Registry. OpSCC patients included anatomical site codes of C01.9 (base of tongue), C02.4 (lingual tonsil), C05.1 (soft palate), C05.2 (uvula), C09 (tonsil), and C10 (oropharynx), following the 3rd Edition of the International Classification of Diseases for Oncology (ICD-O-3) [13]. Additional ICD-O-3 morphological codes of 80,103, 80,203, 80,213, 80,523, 80,703, 80,713, 80,723, 80,733, 80,743, and 80,763 further defined patients for pathological diagnoses of carcinomas and squamous cell carcinomas. Incidence rates of newly diagnosed OpSCC patients were age-adjusted to standardize against the population of BC in 2000. Patient charts were electronically reviewed through the Cancer Agency Information System (CAIS) to collect information on age at diagnosis, sex, primary anatomical site, smoking history, intent and type of primary treatment received, and patient survival 5 years post-diagnosis. Smoking history was categorized as ever-smokers, defined as patients that were former or current smokers, or never-smokers, patients that have never smoked. Patients referred to the BCCA were mainly treated using RT alone or RT with concurrent chemotherapy (CRT). Due to its small number, curative surgical patients referred to the BCCA for adjuvant RT/CRT were excluded from this study. For patient survival, the last follow-up appointment date or the death date, was recorded as the last contact date for alive or deceased patients, respectively. Among deceased patients, the cause of death was categorized as death due to disease or death due to other causes.

A subset of the Study Population with available formalin-fixed, paraffin-embedded (FFPE) tumor biopsy tissues was furthered analyzed as the Study Cohort. The remaining Study Population without FFPE tissues were considered as the General Cohort. The electronic charts of the Study Cohort were further reviewed for TNM staging, adjuvant treatment (if any), and recurrences. The dates for recurrences were collected from reviewing, in descending order of availability, pathology reports, imaging results, or clinical assessments. This study was approved by the University of British Columbia Research Ethics Board (REB #H10–03153).

### Restaging of patients in the study cohort

The Study Cohort consisted of patients that were diagnosed between 2000 and 2008, which used the 7th Edition of the AJCC Head and Neck Cancer Staging Manual for staging. The 8th Edition AJCC Head and Neck Cancer Staging Manual was published in October 2016, and went into effect for cancer cases diagnosed on or after January 1st, 2018. Patients in the Study Cohort were restaged using the AJCC 8th Edition criteria prior to survival analysis. HPV status was first determined by p16 immunohistochemistry (IHC) staining, according to the manufacturer’s protocol, using the E6H4 clone (mouse monoclonal primary antibody, ready-to-use; Ventana Medical Systems, Inc., Tucson, AZ, USA) [12]. Tissue sections, consisted of either cases from whole tissue sections (*n* = 40 cases, 5-μm thick FFPE) or tissue microarray cores (*n* = 204 cases, two 0.6-mm cores per case), were scored at 200-400x magnification by a certified oral pathologist. Positive p16 staining was defined as strong diffuse nuclear and cytoplasmic staining in ≥70% of tumor areas [14].

Following p16 IHC staining, all p16-positive patients in the Study Cohort, were restaged according to the 8th Edition AJCC Head and Neck Staging Manual [12]. Due to limited pre-treatment MRI investigations, the presence of extranodal extension (ENE) could not be reliably attained and therefore, p16-negative patients in the Study Cohort were not restaged.

### Statistical analysis

The Study Cohort was compared to the General Cohort for age at diagnosis, sex, primary anatomical site, smoking history, type of primary treatment received, and 5-year overall survival (OS). Sub-anatomical sites other than the tonsils or base of tongue were grouped together as “other oropharynx”. The continuous variable of age was analyzed using Student’s *t*-test. Fisher’s exact test was used to analyze the remaining categorical variables. Kaplan-Meier (KM) survival analysis was used to estimate the 5-year OS, disease-specific survival (DSS), and locoregional recurrence-free survival (LFS). Date of diagnosis, as based on pathology report, was used as the initial timepoint for time-to-outcome events. For 5-year OS, any cause of death was considered an event. For DSS, death due to the disease, including regional and distant metastasis, was considered an event. Local and regional recurrences were grouped into LFS and the earlier date was used as the endpoint for patients that developed both recurrences at separate instances. Log-rank tests were used to determine the statistical significance between groups. Statistical analysis was conducted using the R software (ver. 3.3.3, R Core Team, Vienna, Austria) and KM survival analysis was conducted using the *survival* package (ver. 2.40–1). Results were considered statistically significant at *p* ≤ 0.05.

## Results

### Burden of OpSCC in British Columbia

**Fig. 1:**
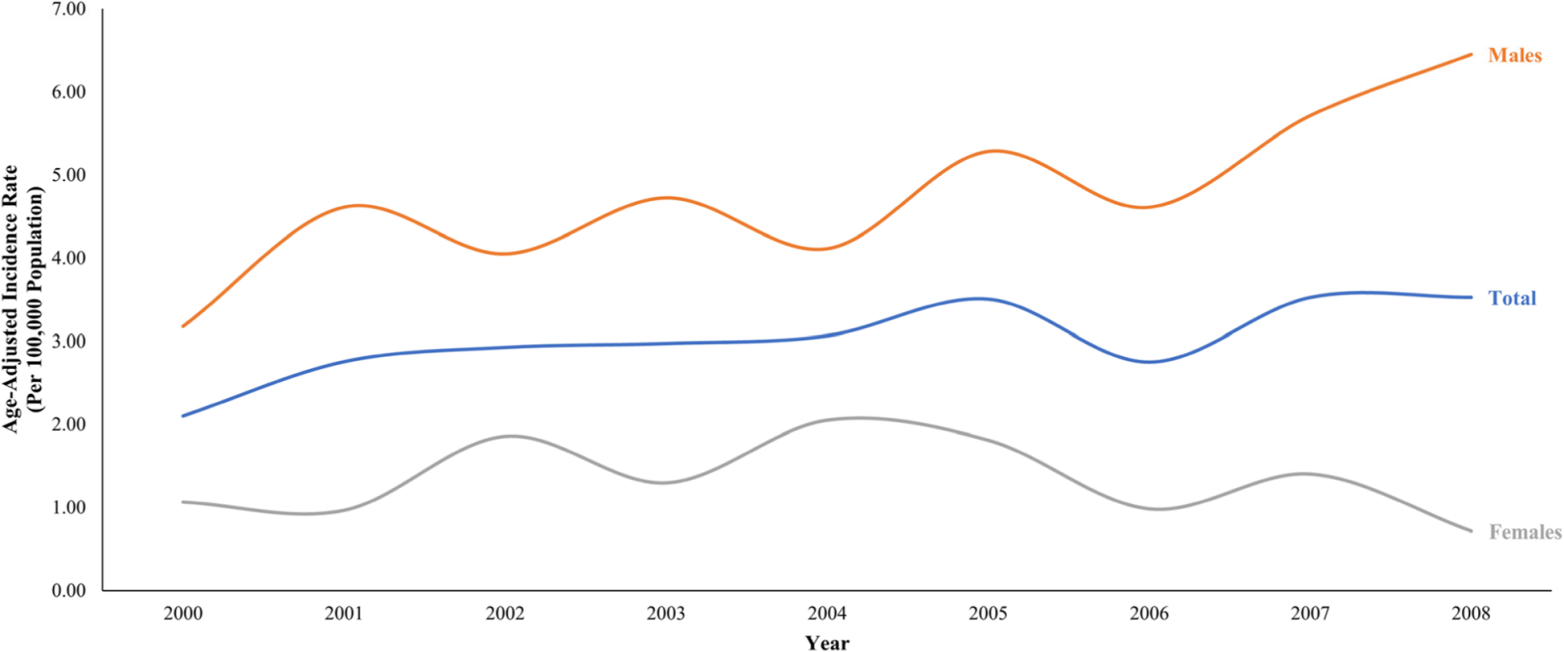
Incidence rate of newly diagnosed oropharyngeal squamous cell carcinoma patients treated with curative intent radiotherapy or chemoradiotherapy in British Columbia between 2000 and 2008. Rates are age-adjusted to standardize against the population of British Columbia in 2000

Based on the inclusion criteria, 1259 patients were identified from the BC Cancer Registry. After initial chart reviews, 288 patients were excluded from further analysis due to not receiving curative treatment (*n* = 198), the treatment prescribed was not primarily RT (*n* = 65) or incomplete data (*n* = 25). A total of 971 patients were included for analysis, representing 77.1% of the total OpSCC cases diagnosed in BC. During this time period, the age-adjusted incidence rate (AAIR) of OpSCC treated with curative intent radiotherapy with or without chemotherapy increased from 2.1 to 3.5 per 100,000 population, with the male AAIR increasing from 3.2 to 6.5 per 100,000, and the female AAIR decreasing from 1.1 to 0.7 per 100,000 (Fig. 1).

### Patient demographics and clinical characteristics between study and general cohorts

Among 971 analyzed patients, 25.1% of the patients (*n* = 244) had available FFPE tissues for p16 IHC staining and referred as the Study Cohort, with the remaining patients (*n* = 727) referred as the General Cohort. Comparison of the two cohorts assessed the representativeness of the Study Cohort (Table 1). Compared to the General Cohort, the Study Cohort had a younger mean age at diagnosis and higher representation of tonsil primary. Radiation was administered at a median dosage of 66 Gy. Among CRT treated patients, Cisplatin was the most commonly administered chemotherapy agent in both General (66.7%, *n* = 198) and Study Cohorts (78.7%, *n* = 85). In the Study Cohort, 21.3% of patients (*n* = 52) developed locoregional recurrence.

**Table 1:** Comparison of demographics and clinical factors between General and Study Cohorts

Variables, no. (%)	General Cohort (***n*** = 727)	Study Cohort (***n*** = 244)	***p***-value	p16-positive (***n*** = 189)	p16-negative (***n*** = 55)	***p***-value
*Age, years (Mean ± SD)*	60.1 ± 10.5	57.3 ± 9.4	**< 0.01**	56.4 ± 9.2	60.3 ± 9.5	**< 0.01**
*Sex*			0.53			0.21
Male	569 (78.3)	186 (76.2)		148 (78.3)	38 (69.1)	
Female	158 (21.7)	58 (23.8)		41 (21.7)	17 (30.9)	
*Anatomical site*^a^			**< 0.01**			**0.03**
Tonsil	314 (43.2)	190 (77.9)		153 (81.0)	37 (67.3)	
Base of tongue	311 (42.8)	48 (19.7)		33 (17.5)	15 (27.3)	
Other oropharynx^b^	98 (13.5)	5 (2.0)		2 (1.1)	3 (5.5)	
*Smoking history*^c^			0.22			**< 0.01**
Never-smoker	137 (18.8)	54 (22.1)		49 (25.9)	5 (9.1)	
Ever-smoker	590 (81.2)	186 (76.2)		137 (72.5)	49 (89.1)	
*Primary treatment*			0.37			0.22
RT	430 (59.1)	136 (55.7)		101 (53.4)	35 (63.6)	
CRT	297 (40.9)	108 (44.3)		88 (46.6)	20 (36.4)	
*5-year survival*			0.06			**< 0.01**
Alive	451 (62.0)	168 (68.9)		142 (75.1)	26 (47.3)	
Died of disease	168 (23.1)	53 (21.7)		36 (19.0)	17 (30.9)	
Died of other cause	108 (14.9)	23 (9.4)		11 (5.8)	12 (21.8)	

*Abbreviations*: *RT* Radiotherapy, *CRT* Concurrent chemoradiotherapy

^a^Unknown primary (*n* = 5)

^b^Includes: Soft palate (*n* = 48), Oropharyngeal wall (*n* = 32), Vallecula (*n* = 14), Uvula (*n* = 8), Anterior surface of epiglottis (*n* = 1)

^c^Unknown smoking status (*n* = 4)

### Restaging the study cohort to 8th edition

The FFPE was stained for p16 IHC and revealed p16-positivity rate of 77.5% (Table 1). Compared to p16-negative patients, p16-positive patients were relatively younger, more likely to have tonsillar primaries, more likely to be never-smokers, and had better 5-year OS. There were no statistically significant differences in terms of sex (*p* = 0.21), and primary treatment (CRT vs. RT alone, *p* = 0.22) between p16-positive and p16-negative tumors.

**Fig. 2:**
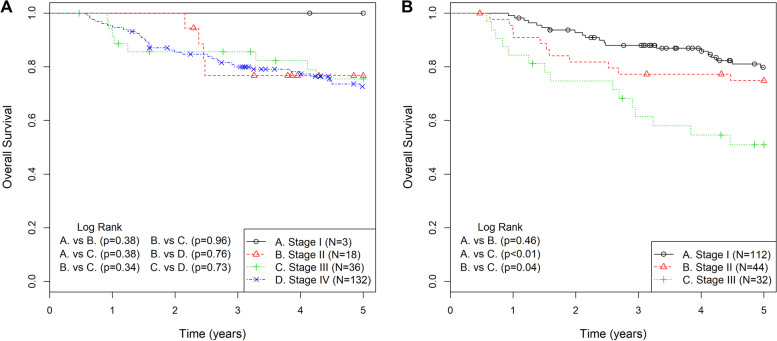
Kaplan-Meier survival and log-rank analysis of oropharyngeal squamous cell carcinoma patients for 5-year overall survival. P16-positive patients were staged using the 7th Edition (**A**) and the 8th Edition (**B**) of the American Joint Committee on Cancer Staging Manual

With available p16 status, p16-positive (*n* = 189) patients were restaged according to the 8th Edition of the AJCC Head and Neck Staging Manual (Additional File 1) [12]. In our Study Cohort, 98.4% of p16-positive patients were down staged, such that 88.9% of p16-positive patients that were either stage III or IV (7th Edition), have been reduced to 16.9% as stage III (8th Edition). Overall Kaplan-Meier survival analysis for p16-positive patients, based on stage according to the 7th and 8th Edition, is presented in Fig. 2A and B, respectively. The 8th Edition was superior to the 7th Edition in stratifying 5-year OS. There was no statistically significant difference in 5-year OS between stages II, III, and IV (72.8, 65.0 and 66.8%, respectively, *p* = 0.91) based on the 7th Edition. In contrast, the 8th Edition stage III patients had significantly poorer 5-year OS (51.0%) when compared to both stage I (79.8%, *p* < 0.01) and stage II (74.9%, *p* = 0.04). The 55 p16-negative patients could not be restaged based on the 8th Edition because of the inconsistent use of MRI to determine ENE status.

### Association of Survival with treatment modality

**Fig. 3:**
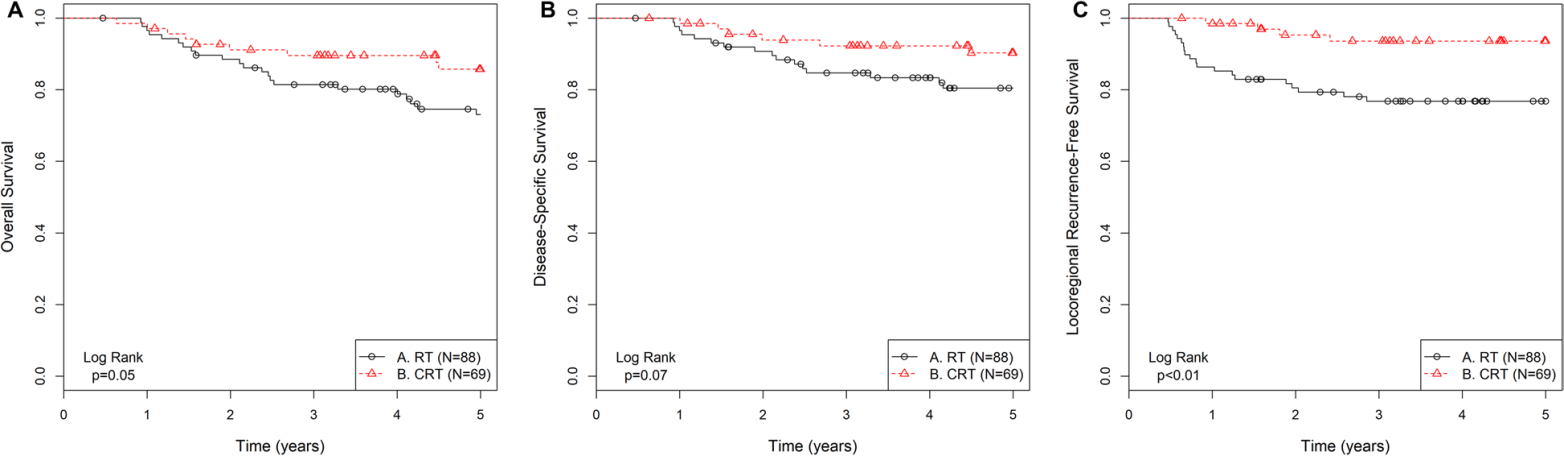
Kaplan-Meier survival and log-rank analysis of treatment for early-stage, p16-positive oropharyngeal squamous cell carcinoma patients for 5-year overall survival (**A**), disease-specific survival (**B**), and locoregional recurrence-free survival (**C**). Patients were assessed using the 8th Edition for early-stages of the American Joint Committee on Cancer Head and Neck Staging Manual. Abbreviations: RT: Radiotherapy; CRT: Concurrent chemoradiotherapy

Outcomes for 5-year OS, DSS, and LFS were compared based on 8th Edition staging and treatment modality of either CRT or RT alone (Fig. 3). Interestingly, a comparison of early-stage patients (*n* = 157) found that those who received CRT (*n* = 69) than RT (*n* = 88) had improved 5-year OS (85.8% vs. 73.1%, *p* = 0.05), DSS (90.3% vs. 80.4%, *p* = 0.07), and LFS (93.6% vs. 76.9%, *p* < 0.01) (Fig. 3A-C). Stage III patients (*n* = 32) who received CRT (*n* = 19) than RT (*n* = 13) also had improved 5-year OS (56.7% vs. 43.3%, *p* = 0.27), DSS (60.0% vs. 47.2%, *p* = 0.31) and LFS (72.7% vs. 68.4%, *p* = 0.53), but none reaching statistical significance (Additional File 2).

## Discussion

The current study retrospectively reviewed the population based incidence and management of OpSCC treated with curative intent radiotherapy with or without chemotherapy, in BC between 2000 and 2008. BC is a province of Canada with a population of ~ 5 million, where all newly diagnosed OpSCC are registered at a central intake and treated at one of six cancer agencies. While tobacco consumption continues to decline in BC [15], the AAIR of OpSCC over the study period has increased from 2.1 to 3.5 per 100,000, mostly comprised of males patients. This trend is witnessed worldwide and is attributed to HPV-induced oncogenesis [9]. Consistent with previous literature, the p16-positive patients, compared to p16-negative, had a higher proportion of males, younger age, decreased tobacco use, and a drastically improved 5-year OS [9, 16–18].

Since the introduction of the 8th Edition AJCC Head and Neck Staging Manual, studies have evaluated the improved prognostic predictability of this new staging criteria [19–21]. Indeed, the provincial-wide data presented here, reveals no significant difference in OS for stage I to IV patients, based on the AJCC 7th Edition. However, p16-positive patients restaged using the 8th Edition revealed a significant difference of 5-year OS when comparing Stage III (51.0%) to either Stage I (79.8%) or Stage II (74.9%). In this study, patients were not routinely stained for p16 upon initial diagnosis and therefore, management was not influenced by HPV status.

Restaging the present cohort according to the 8th Edition found a significant stage redistribution. Notably, early-stage disease patients who received CRT than RT alone revealed an improved DSS, and significant improvement in OS and LFS. The addition of chemotherapy to RT appears play some role to improve OS even for early-stage disease. A study from the National Cancer Database (NCDB) restaged patients from the 7th to 8th Edition, reported a similar 3-year OS of stage I patients compared to our stage I patient population of 90.3% vs. 91.3%, respectively. The NCDB study also revealed a statistically improved OS of stage I OpSCC treated with CRT vs. RT (91.3% vs. 80.6%), similar to our findings [21]. A retrospective review of ~ 280 node positive AJCC 8th stage I p16-positive OpSCC was designed to identify adverse features affecting prognosis. Using multivariate analysis, the addition of chemotherapy, which was received by 70% of stage I patients, was one of the four independent variables in predicting disease-free survival [22]. Importantly, treatment in the aforementioned studies, including the presented case series, was nonrandomly assigned, and therefore the perceived improvement in DFS and OS by the addition of systemic therapy could be biased.

Studies have demonstrated the inferiority of CRT with Cetuximab as compared to Cisplatin-based CRT for OS and locoregional control [23, 24]. Our study supported the use of Cisplatin-based concurrent CRT. In the RTOG 1016 study [23], 50.6% of stage I patients (*n* = 407/805) showed Cetuximab as inferior to Cisplatin. Similarly, the De-ESCALaTE HPV phase III trial [24] revealed a decrease in OS and an increase in recurrence rates in those randomized to Cetuximab vs. standard Cisplatin. In the NRG-HN002 phase II trial [25], patients receiving a reduced dose of curative RT without concurrent weekly Cisplatin experienced higher locoregional failure (9.5% vs. 3.3% respectively, *p* = 0.02) and worse progression free survival, compared to those receiving the same radiation therapy regimen with weekly Cisplatin. In our Study Cohort, early-stage patients who received CRT, rather than RT alone, had significantly improved 5-year LFS and OS. None of our Study Cohort patients received Cetuximab for comparison with contemporary studies. Whether Cisplatin is superior to other chemotherapy agents for improving patient outcome warrants further investigation.

Studies using de-escalation strategies are appealing for the p16-positive cohort. These strategies include concomitant CRT with possibility of less toxic chemotherapy regimens [23, 24], removal of chemotherapy (RT alone) [26], reducing the dose of RT [25, 27], and trans-oral surgery [28]. Our Study Cohort patients received the standard of care for RT, i.e., radiation dosage at 70 Gy, and the benefit of concurrent chemotherapy was still observed among our early-stage patients who had received CRT. Therefore, de-escalation studies that aim to reduce the RT dosage should be cautioned if concurrent chemotherapy is not administered, even for early-stage OpSCC.

One of the study limitation is that the study period predated the initiation of a Trans-Oral Robotics program (NCT01590355) [29] and therefore precluded surgery as a treatment modality for OpSCC. The application of trans-oral surgery in the context of treatment de-escalation has been studied prospectively in only one phase II randomized trial, but had its primary endpoint as the MD Anderson Dysphagia index and not oncologic outcomes [29]. Several prospective trials are currently underway with results pending [28, 30].

Other limitations of this study include the retrospective sample set with an inherent selection bias and an unequal stage distribution with limited numbers in some sub-groups precluding adequate power. Furthermore, the lack of controlling confounding clinical variables such as comorbidities and tobacco pack-years limits the findings and therefore the addition of chemotherapy to radiotherapy may not independently improve the outcome of early-stage p16-positive OpSCC patients. As well, it is possible that patients’ inherent health status precluded the addition of chemotherapy and therefore only suitable for RT alone and as a result may have correlated with their decreased survival. The Study Cohort was limited to the availability of FFPE on which p16 staining was performed, but nevertheless represented 25.1% of the total 971 analyzed patients. This resulted in a larger proportion of tonsillar subsite (vs. base of tongue) compared to that of the General Cohort, as this subsite is more amenable to biopsy with enough tissue for further HPV genotyping.

## Conclusion

OpSCC has increased significantly in male patients in BC with a vast majority mediated by HPV, as determined by p16 IHC staining. Early-stage patients revealed a significant improvement in OS for those treated with CRT vs. RT alone. This study lends further support to the utility of the addition of chemotherapy to radiotherapy to improve OS, even for early-stage I/II p16-positive OpSCC. Further prospective study is required to investigate the oncologic safety of treatment de-escalation even for early-stage disease.

## Supplementary Information

**Additional file 1.** Restaging p16-positive OpSCC patients from 7th to 8th Edition of the AJCC Cancer Staging Manual.

**Additional file 2.** Kaplan-Meier survival and log-rank analysis of treatment for stage III, p16-positive oropharyngeal squamous cell carcinoma patients for 5-year overall survival (A), disease-specific survival (B), and locoregional recurrence-free survival (C). Patients were assessed using the 8th Edition of the American Joint Committee on Cancer Head and Neck Staging Manual. Abbreviations: RT: Radiotherapy; CRT: Concurrent chemoradiotherapy

## Data Availability

The datasets used and/or analysed during the current study are not made publicly available due to ethics restrictions. However, the datasets are available from the corresponding author upon reasonable request.

## Abbreviations

OpSCC: Oropharyngeal squamous cell carcinoma
HR-HPV: High-risk human papillomavirus
AJCC: American Joint Committee on Cancer
BC: British Columbia
BCCA: British Columbia Cancer Agency
CAIS: Cancer Agency Information System
RT: Radiation therapy
CRT: Concurrent chemoradiotherapy
FFPE: Formalin-fixed, paraffin-embedded
IHC: Immunohistochemistry
ENE: Extranodal extension
KM survival analysis: Kaplan-Meier survival analysis
OS: Overall survival
DSS: Disease-specific survival
LFS: Locoregional recurrence-free survival
AAIR: Age-adjusted incidence rate
NCDB: National Cancer Database
