# Supplementary material for: Combined chemoradiotherapy showed improved outcome with early-stage HPV-positive oropharyngeal cancers
Source: BMC Cancer. 2022 May 7;22:513. doi: 10.1186/s12885-022-09515-2 (PMC9077931; doi:10.1186/s12885-022-09515-2)
Supplement: Supplementary file 1 — Additional file 1. Restaging p16-positive OpSCC patients from 7th to 8th Edition of the AJCC Cancer Staging Manual. [file 12885_2022_9515_MOESM1_ESM.docx]

**Additional File 1.** Restaging p16-positive OpSCC patients from 7^th^ to 8^th^ Edition of the AJCC Cancer Staging Manual.

| **TNM stage** | **7^th^ Edition**  **No. (%)** | **8^th^ Edition**  **No. (%)** |
| --- | --- | --- |
|  |  |  |
| *Tumor stage* |  |  |
| TX | 2 (1.1) | 0 |
| T0 | 1 (0.5) | 3 (1.6) |
| T1 | 53 (28.0) | 53 (28.0) |
| T2 | 82 (43.4) | 82 (43.4) |
| T3 | 37 (19.6) | 37 (19.6) |
| T4 | 14 (7.4) | 14 (7.4) |
|  |  |  |
| *Nodal stage* |  |  |
| N0 | 28 (14.8) | 28 (14.8) |
| N1 | 32 (16.9) | 119 (63.0) |
| N2 | 110 (58.2) | 23 (12.2) |
| N3 | 19 (10.1) | 19 (10.1) |
|  |  |  |
| *AJCC staging* |  |  |
| I | 3 (1.6) | 112 (59.3) |
| II | 18 (9.5) | 45 (23.8) |
| III | 36 (19.0) | 32 (16.9) |
| IV | 132 (69.8) | 0 |
|  |  |  |

Abbreviation: AJCC: American Joint Committee on Cancer
