# Supplementary material for: Combined chemoradiotherapy showed improved outcome with early-stage HPV-positive oropharyngeal cancers
Source: BMC Cancer. 2022 May 7;22:513. doi: 10.1186/s12885-022-09515-2 (PMC9077931; doi:10.1186/s12885-022-09515-2)
Supplement: Supplementary file 2 — Additional file 2. Kaplan-Meier survival and log-rank analysis of treatment for stage III, p16-positive oropharyngeal squamous cell carcinoma patients for 5-year overall survival (A), disease-specific survival (B), and locoregional recurrence-free survival (C). Patients were assessed using the 8th Edition of the American Joint Committee on Cancer Head and Neck Staging Manual. Abbreviations: RT: Radiotherapy; CRT: Concurrent chemoradiotherapy [file 12885_2022_9515_MOESM2_ESM.docx]

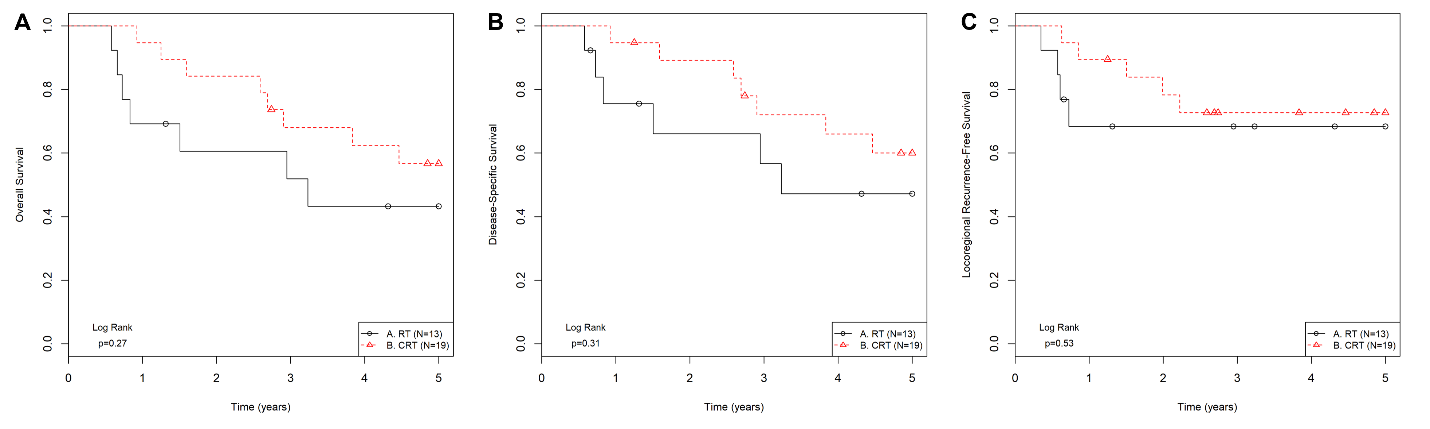


**Additional File 2.** Kaplan-Meier survival and log-rank analysis of treatment for stage III, p16-positive oropharyngeal squamous cell carcinoma patients for 5-year overall survival (A), disease-specific survival (B), and locoregional recurrence-free survival (C). Patients were assessed using the 8^th^ Edition of the American Joint Committee on Cancer Head and Neck Staging Manual. Abbreviations: RT: Radiotherapy; CRT: Concurrent chemoradiotherapy
